# Repair for the Anomalies of Ventriculoarterial Connection with Ventricular Septal Defect and Left Ventricular Outflow Tract Obstruction

**DOI:** 10.1186/1749-8090-10-S1-A83

**Published:** 2015-12-16

**Authors:** Woong-Han Kim, Jeong Ryul Lee, Yong Jin Kim

**Affiliations:** 1 Department of Thoracic and Cardiovascular Surgery, Seoul National University Hospital, Seoul, Korea

## Background/Introduction

Anomalies of ventriculoarterial connection with ventricular septal defect (VSD) and left ventricular outflow tract (LVOT) obstruction such as transposition of the great arteries, double-outlet right ventricle, double-outlet left ventricle, and Taussig-Bing anomaly had a wide variety of spectrum, and several operative techniques have been performed according to diverse anatomical characteristics without standard operative selection guidelines.

## Aims/Objectives

This study was undertaken to compare the outcomes of the Lecompte procedure and Rastelli repair in anomalies of ventriculoarterial connection with VSD and LVOT obstruction.

## Method

Over a 35-year period (1979- 2014), 95 patients underwent complete repair for anomalies of ventriculoarterial connection with VSD and LVOT obstruction. Fifty patients (52.6%) underwent the Lecompte modification, and median age and weight were 1.95 years (range: 0.30-12.48) and 10.1 kg (range: 5.7-35). Forty five patients (47.4%) underwent the Rastelli operation, and median age and weight were 3.25 years (range: 0.36-46.15) and 13.0 kg (range: 5.9-55).

## Results

There were thirteen deaths after complete repair. Twenty three (46.0%) patients in the Lecompte group underwent reoperation, and thirty three (73.3%) in the Rastelli group underwent reoperation. Freedom from reoperation was 25.2 ± 9.4% at 25 years in the Lecompte group and 5.5 ± 4.8% at 27 years in the Rastelli group (p = 0.01). Freedom from reoperation for right ventricular outflow tract (RVOT) obstruction was 49.6 ± 9.0% at 25 years in the Lecompte group and 6.8 ± 5.8% at 27 years in the Rastelli group (p = 0.01). Freedom from reoperation for LVOT obstruction was 88.5 ± 5.4% at 25 years in the Lecompte group and 60.7 ± 10.4% at 33 years in the Rastelli group (p = 0.01).

## Discussion/Conclusion

The Lecompte procedure and Rastelli repair provide satisfactory results at long-term follow-up. Substantial late morbidity is more associated with RVOT obstruction, and LVOT obstruction in Rastelli repair rather than Lecompte procedure.

